# 968. Leveraging On-Demand Digital Education to Increase ID Specialists’ Knowledge and Confidence on Appropriate Probiotic Use for CDI Prevention

**DOI:** 10.1093/ofid/ofab466.1163

**Published:** 2021-12-04

**Authors:** Allison Armagan, Simi Thomas Hurst

**Affiliations:** 1 Medscape, New York, New York; 2 Medscape Education, Oxford, NJ

## Abstract

**Background:**

Probiotics are increasingly being used in healthcare. As the number of probiotic options and their potential uses increase, it has become more challenging to make an informed selection for a given disease state. This study assessed the ability of digital education to improve Infectious Disease (ID) specialists’ knowledge regarding the use of probiotics in preventing *Clostridioides difficile* infection (CDI) and antibiotic-associated diarrhea (AAD).

**Methods:**

A CME/ABIM MOC certified, educational program featuring a panel of 3 expert ID faculty was developed. The program sought to clarify the role of different probiotic strains in the prevention or treatment of different disease states. Educational effectiveness was assessed with a repeated-pairs pre-/post-assessment study design; each individual served as his/her own control. A chi-square test assessed changes pre- to post-assessment. *P* values < 0.05 are statistically significant. Effect sizes were evaluated using Cramer’s V (< 0.05 modest; 0.06-0.15 noticeable effect; 0.16-0.26 considerable effect; > 0.26 extensive effect). The activity launched on a website dedicated to continuous professional development. Data for this matched-learner analysis were collected through 09/09/20.

**Results:**

To date, 7122 HCPs, including 5068 physicians, have participated in the activity. Data from the subset of ID specialists (n=235) who answered all pre-/post-assessment questions during the initial study period were analyzed. Following activity participation, significant improvements were observed in the proportion of ID specialists who answered assessment questions correctly (47% pre vs 69% post; *P* < .0001; V=.22). Improvements were also observed in several specific areas of assessment (Table) and confidence in their ability to select a probiotic-based on strain- and disease-specific efficacy (36% increase).

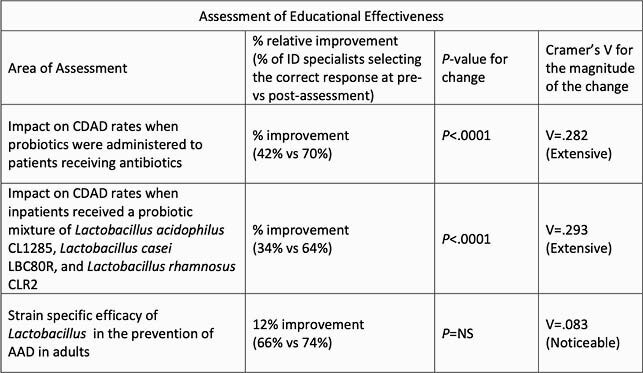

**Conclusion:**

Participation in this online educational program significantly improved ID specialists’ understanding of the interplay between strain- and disease-specificity in the context of probiotics. These findings highlight the potential for well-designed online education to positively impact physicians’ knowledge and confidence

**Disclosures:**

**All Authors**: No reported disclosures

